# Acute bilateral suppurative parotitis following treatment of acute decompensated heart failure with noninvasive positive-pressure ventilation

**DOI:** 10.1093/omcr/omac057

**Published:** 2022-06-23

**Authors:** Kenta Yasuoka, Seika Sai, Nana Tsukahara, Kenji Machiki, Takayoshi Yamanouchi

**Affiliations:** Department of Cardiology, Hitachinaka General Hospital, Hitachinaka, Japan; Department of Cardiology, Hitachinaka General Hospital, Hitachinaka, Japan; Department of Otolaryngology, Hitachinaka General Hospital, Hitachinaka, Japan; Department of Otolaryngology, Hitachinaka General Hospital, Hitachinaka, Japan; Department of Cardiology, Hitachinaka General Hospital, Hitachinaka, Japan

## Abstract

We present a case of acute bilateral suppurative parotitis in an elderly patient with acute heart failure who was hospitalized and treated with noninvasive positive-pressure ventilation. In this case, sepsis due to infection occurred, leading to severe illness.

## CASE PRESENTATION

**Figure 1:**
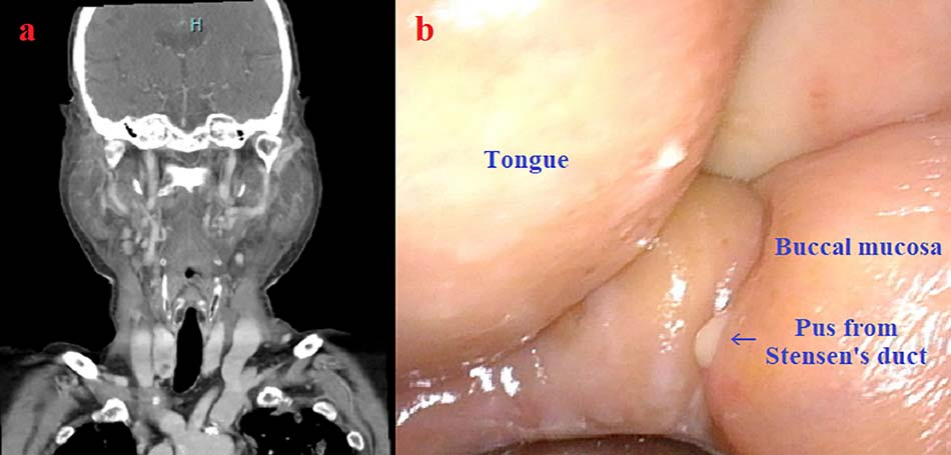
(a) Marked swelling of the bilateral parotid glands shown by computed tomography scan; (b) laryngoscopic view of pus drainage from Stensen’s duct.

A 92-year-old woman presented to the emergency department with dyspnea. She was diagnosed with acute decompensated heart failure and admitted to the intensive care unit. Chest radiography revealed severe bilateral perihilar alveolar edema. She was treated with vasodilators and noninvasive positive-pressure ventilation (NIPPV) by bilevel positive airway pressure with inspiratory and expiratory positive airway pressure of 14 and 6 cmH_2_O, respectively. NIPPV was discontinued on the 3rd day after admission. However, painful swelling of the bilateral parotids developed on the following day. Computed tomography showed diffusely enlarged bilateral parotids (Fig. 1a). A laryngoscopy showed purulent drainage from Stensen’s duct (Fig. 1b). Suppurative parotitis was diagnosed, and antibiotics were initiated. Methicillin-susceptible *Staphylococcus aureus* was isolated from both the blood and drainage cultures. This led to sepsis, which triggered another exacerbation of heart failure, thereby, making treatment difficult and ultimately fatal.

Acute suppurative parotitis is a rare infection caused by the invasion of oral commensals into Stensen’s duct due to salivary stasis, with typical symptoms such as the acute onset of parotid swelling and pain. Diagnosis is based on drainage from Stensen’s duct and imaging results, with *S. aureus* reported to be the most common causative bacteria [1]. NIPPV-associated parotitis is thought to be due to retrograde air entry from the oral cavity and obstruction of Stensen’s duct by positive pressure ventilation, and it can be unilateral or bilateral [2, 3]. The incidence is not yet known. When salivary flow is stagnant due to dehydration or decreased oral intake, there is a predisposition to the development of parotitis [1], and the use of NIPPV in such a circumstance may increase the risk. As presented in this case, suppurative parotitis can be fatal; therefore, prompt diagnosis and treatment are required if swelling of the parotid gland occurs during NIPPV use.

## CONFLICT OF INTEREST STATEMENT

The authors have reported that they have no relationships relevant to the contents of this paper to disclose.

## FUNDING

The authors received no specific grants for this research from any funding agency in the public, commercial or not-for-profit sectors.

## ETHICAL APPROVAL

No ethical approval is required for case report in our centre.

## CONSENT

The authors confirm that written consent for the submission and publication of this case was obtained from the patient’s family.

## GUARANTOR

Seika Sai.
